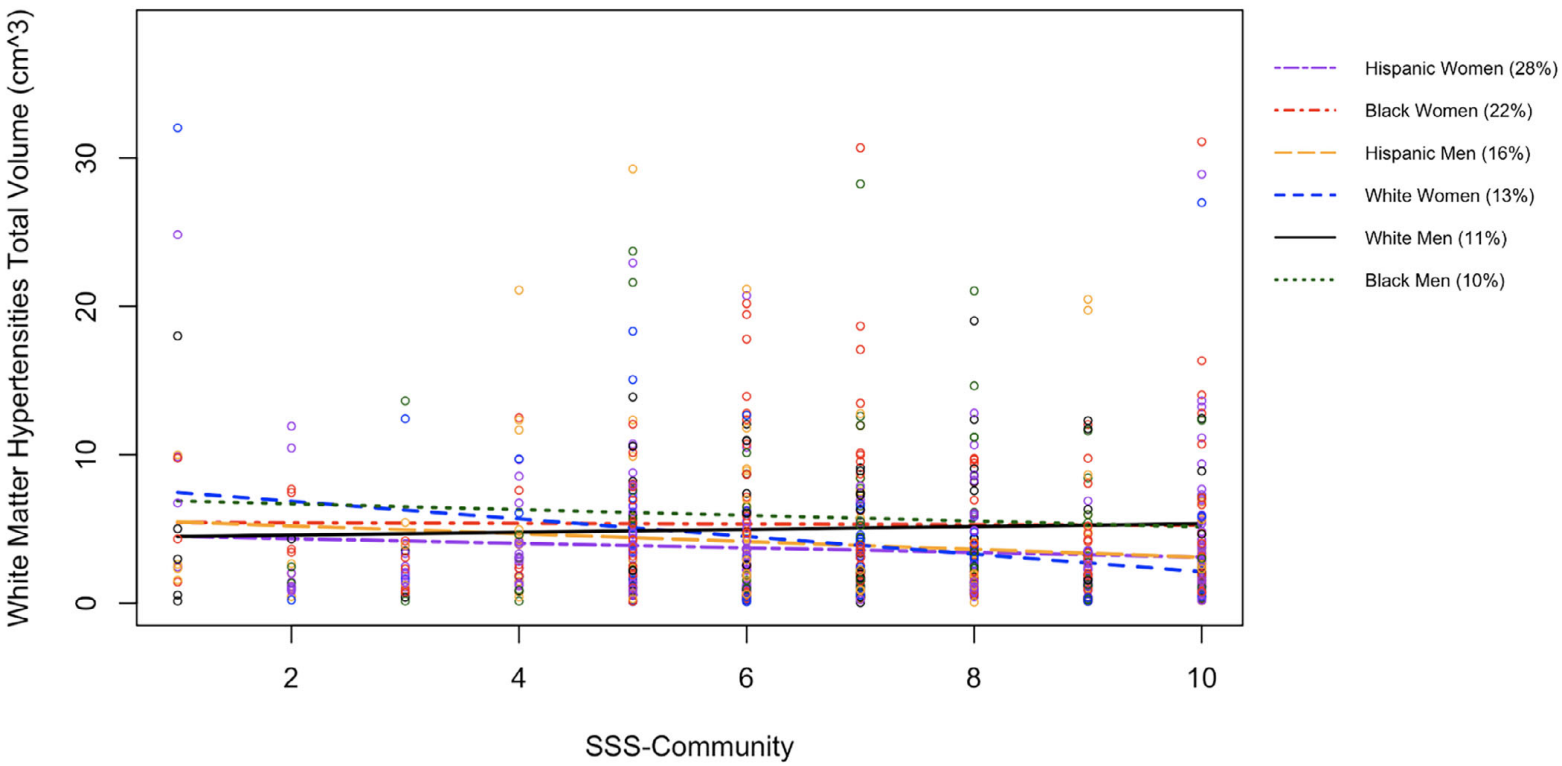# Subjective Social Status, Brain Health, and Cognitive Reserve Across Race/Ethnicity and Sex/Gender

**DOI:** 10.1002/alz.087985

**Published:** 2025-01-09

**Authors:** Kiana A. Scambray, Monica E Nelson, Emily P. Morris, Jordan D Palms, Lauren Taylor, Ketlyne Sol, Nicole Schupf, Adam M. Brickman, Jennifer J. Manly, Laura B. Zahodne

**Affiliations:** ^1^ University of Michigan, Ann Arbor, MI USA; ^2^ Columbia University Irving Medical Center, New York, NY USA; ^3^ Taub Institute for Research on Alzheimer’s Disease and the Aging Brain, New York, NY USA

## Abstract

**Background:**

Subjective social status in the US (SSS) is related to physical, mental, and cognitive health independent of socioeconomic status, yet few studies have assessed SSS in one’s community or examined how SSS may function differentially across the intersection of race and gender. This study aimed to assess the relationships between SSS‐US, SSS‐community, brain health, and cognitive reserve utilizing an intersectional lens to extend the literature on social determinants of Alzheimer’s disease and related dementia (ADRD) risk.

**Methods:**

Participants were 867 older adults from the Washington Heights‐Inwood Columbia Aging Project (WHICAP). SSS relative to the US and one’s community were based on the MacArthur Scale of SSS. Brain health was operationalized as total volume of white matter hyperintensities (WMH), total gray matter volume, total hippocampal volume, and mean cortical thickness across nine AD signature regions. Cognitive reserve was operationalized as residual variance in an episodic memory composite after accounting for all brain health measures. Separate multiple linear regressions assessed unique relationships between the two SSS predictors and the brain health and cognitive reserve outcomes while adjusting for sociodemographics. Multiplicative interaction terms examined whether associations involving SSS were moderated by sex/gender, race/ethnicity, and their intersection.

**Results:**

There were no relationships between SSS‐community or SSS‐US and brain health across the full sample. Among White women, higher SSS‐community was associated with lower WMH (B = ‐0.33, p = 0.047). Among Black men, higher SSS‐community was associated with lower hippocampal volume (B = ‐0.31, p = 0.04). In sensitivity analyses, higher SSS‐community was associated with lower hippocampal volume among older (B = ‐0.30, p = 0.048), but not younger (B = ‐0.21, p = 0.37) Black men. Neither SSS‐US nor SSS‐community was associated with cognitive reserve.

**Conclusions:**

This study highlights the value of assessing community standing in addition to US standing to understand social determinants of ADRD risk, as community standing may be more predictive of brain health. From an intersectional lens, community standing may be most strongly related to cerebrovascular health among White women, who appeared more sensitive to both low and high community standing. Counterintuitive findings among Black men likely reflect selective survival. Further research should explore longitudinal relationships between community standing and brain health.